# Examining emotion regulation in binge-eating disorder

**DOI:** 10.1186/s40479-021-00166-6

**Published:** 2021-10-08

**Authors:** Anna Walenda, Barbara Kostecka, Philip S. Santangelo, Katarzyna Kucharska

**Affiliations:** 1 The Institute of Psychology, Cardinal Stefan Wyszynski University, Warsaw, Poland; 2 II Department of Psychiatry, Medical University of Warsaw, Warsaw, Poland; 3 Mental mHealth Lab, Karlsruhe Institute of Technology, Karlsruhe, Germany

**Keywords:** Binge-eating disorder, BED, Emotion regulation, Maladaptive strategies, Cognitive strategies

## Abstract

**Background:**

Inefficient mechanisms of emotional regulation appear essential in understanding the development and maintenance of binge-eating disorder (BED). Previous research focused mainly on a very limited emotion regulation strategies in BED, such as rumination, suppression, and positive reappraisal. Therefore, the aim of the study was to assess a wider range of emotional regulation strategies (i.e. acceptance, refocusing on planning, positive refocusing, positive reappraisal, putting into perspective, self-blame, other-blame, rumination, and catastrophizing), as well as associations between those strategies and binge-eating-related beliefs (negative, positive, and permissive), and clinical variables (eating disorders symptoms, both anxiety, depressive symptoms, and alexithymia).

**Methods:**

Women diagnosed with BED (*n* = 35) according to the DSM-5 criteria and healthy women (*n* = 41) aged 22–60 years were assessed using: the Eating Attitudes Test-26, the Eating Beliefs Questionnaire-18, the Hospital Anxiety and Depression Scale, the Toronto Alexithymia Scale-20, the Cognitive Emotion Regulation Questionnaire, and the Difficulties in Emotion Regulation Scale. Statistical analyses included: Student t - tests or Mann–Whitney U tests for testing group differences between BED and HC group, and Pearson’s r coefficient or Spearman’s rho for exploring associations between the emotion regulation difficulties and strategies, and clinical variables and binge-eating-related beliefs in both groups.

**Results:**

The BED group presented with a significantly higher level of emotion regulation difficulties such as: nonacceptance of emotional responses, lack of emotional clarity, difficulties engaging in goal-directed behavior, impulse control difficulties, and limited access to emotion regulation strategies compared to the healthy controls. Moreover, patients with BED were significantly more likely to use maladaptive strategies (rumination and self-blame) and less likely to use adaptive strategies (positive refocusing and putting into perspective). In the clinical group, various difficulties in emotion regulation difficulties occurred to be positively correlated with the level of alexithymia, and anxiety and depressive symptoms. Regarding emotion regulation strategies, self-blame and catastrophizing were positively related to anxiety symptoms, but solely catastrophizing was related to the severity of eating disorder psychopathology.

**Conclusions:**

Our results indicate an essential and still insufficiently understood role of emotional dysregulation in BED. An especially important construct in this context seems to be alexithymia, which was strongly related to the majority of emotion regulation difficulties. Therefore, it might be beneficial to pay special attention to this construct when planning therapeutic interventions, as well as to the maladaptive emotion regulation strategies self-blame and catastrophizing, which were significantly related to BED psychopathology.

**Supplementary Information:**

The online version contains supplementary material available at 10.1186/s40479-021-00166-6.

## Introduction

Nowadays, binge-eating disorder (BED) is one of the most common diseases among feeding and eating disorders in DSM-5 classification (Diagnostic and Statistical Manual of Mental Disorders, 5th Edition) [1]. Its lifetime prevalence among the general population from United States is estimated to about 2,03% [2]. BED manifests by recurrent episodes of eating large amounts of food in a discrete period (e. g. within 2 h), while experiencing a sense of lack of control [1]. The binge-eating episodes are characterized by: (a) rapid pace of eating, (b) eating until feeling extremely full, (c) eating without a feeling of physical hunger, (d) feeling guilty and disgusted about oneself afterwards [1]. Unlike bulimia nervosa (BN), inappropriate compensatory behaviors, such as purging, do not occur in BED [1]. A recent study found that some specific metacognitive beliefs about food and eating may be a maintaining factor in binge-eating symptoms [3]. Namely, three types of beliefs could be distinguished: negative beliefs (thoughts of having no control over one’s eating), positive beliefs (beliefs that eating improves one’s mood), and permissive beliefs (beliefs that one should allow oneself to binge-eat) [3]. Furthermore, the dysfunctional mechanisms of emotion regulation seem to be one of the most problematic issues of the clinical picture of BED [4]. Emotional regulation is a multidimensional construct that comprises all processes with which a person monitors, assesses, and modifies his or her emotional states [5]. It can be regarded as the ability to inhibit impulsive, dysfunctional reactions to strong emotions regardless of their valence or refocusing attention while experiencing intense emotions or self-restraining physiological stimulation that accompanies them [6].

The inability to regulate emotions adaptively seems to be important not only for the development but also for sustaining binge-eating [7, 8]. There are several models in which researchers have tried to explain these associations. One of the oldest models, the escape from self-awareness model by Heatherton and Baumeister [9], depicted overeating as an attempt to escape the aversive perception of oneself by narrowing the field of attention, which relates to the use of evasive coping strategies [10]. Fairburn’s transdiagnostic theory of eating disorders [11] raised an issue that intolerance of both positive and negative mood presents in some patients who overeat significantly hinders the effects of standard treatment (requiring additional interventions). However, the association between overeating episodes and the presence of negative emotions is particularly highlighted in the growing body of literature. The affect regulation model described by Polivy and Herman [12] sees the function of binges in alleviating negative affect, which secondarily reinforces a maladaptive coping means that becomes increasingly learned and automated. This model stays in line with extensive scientific literature, as follows: the systematic review by Leehr and coworkers [13], the meta-analysis by Cardi et al. [14] in conjunction with a significant part of the (electronic) diary studies [15–18]. According to the above-mentioned diary studies [15–18], negative affect in people with BED often becomes a trigger for overeating, which is not the case in people without this diagnosis, even in the case of obesity. However, Munsch et al. [18] state that it is not so much the occurrence or accumulation of negative emotions that is important, but their rapid increase, causing considerable tension, and consequently the breakdown of insufficiently efficient processes of regulating emotions. Giel et al. [19], on the other hand, stress the role of positive emotions associated with food consumption and the pleasure it may provide. Thus, making it more difficult to face impulses, which is particularly difficult in stressful situations [20]. There is a lack of consistency among empirical results on the impact of overeating on emotions and mood. According to some results, the affective state improves [15, 18], while, according to others, it worsens [10, 16, 21] after engaging in binge-eating.

Researchers posit that especially in case of coping with aversive emotions, overeating appears a consequence of a lack of more adaptive strategies of emotion regulation [7]. A wide range of possible strategies can be divided into adaptive and maladaptive ones, whereas the latter are related to psychopathology [22]. Examples of adaptive emotional regulation strategies are: cognitive reappraisal (i.e. attempts to change emotions by changing the interpretation of a given situation) [23], acceptance of the situation, refocusing on planning actions to deal with it, positive refocusing (thinking of other, pleasant matters instead of the negative event), positive reappraisal (treating a difficult situation as an opportunity for development), or putting into perspective (associating the relativity of a difficult event with other events) [24]. Maladaptive strategies of coping with difficult emotions would be, for example, overeating, [25], blaming oneself for a given situation or blaming others, catastrophic thinking, ruminating (persistent remembrance of a negative event and its consequences), [26] or suppression [22]. Rumination is a characteristic strategy engaged in the psychopathology of eating disorders, [22, 26–28] including binge-eating [22, 27]. Moreover, some studies found that individuals with BED tend to reappraise their emotions less in comparison to healthy controls [29, 30]. However, there is a paucity of research on certain emotion regulation strategies applied by BED patients.

The aim of the present study was to identify various emotion regulation difficulties in individuals diagnosed with BED in comparison to a healthy control group. Previous research (e. g [31, 29].) focused mainly on a very few emotion regulation strategies in BED, such as rumination, suppression, and positive reappraisal. Therefore, little is known so far about the other strategies. To address this gap in the literature, this is the first study, to the authors’ best knowledge, which allowed to assess such a wide range of emotion regulation strategies (including self-blame, catastrophizing, others-blame, putting into perspective, acceptance, positive refocusing, refocus on planning, positive reappraisal), used by BED patients in comparison to healthy controls. Moreover, this study aimed to analyze a spectrum of potential correlative interactions between applied strategies and difficulties in emotion regulation, and clinical variables (severity of eating disorders, severity of anxiety and depression symptoms, and level of alexithymia) in both subject groups, as depression, anxiety, and alexithymia are often comorbid with BED and determine its course [32, 33]. To the best of authors’ knowledge, this is also the first study exploring associations between binge- eating-related beliefs (positive, negative, and permissive) and various emotional regulation strategies and difficulties in individuals diagnosed with BED. This is especially important as these beliefs seems to be maintaining factors in BED [3].

In line with existing literature, we hypothesized that: (1) patients with BED report greater difficulties in emotion regulation than healthy controls, (2) patients with BED use more maladaptive strategies and less adaptive strategies than healthy controls, (3) various difficulties in emotion regulation and using of maladaptive strategies in BED group are positively correlated with the severity of eating disorders symptoms, depression, anxiety, and alexithymia, whereas (4) the use of adaptive strategies is negatively correlated with the severity of eating disorders symptoms, depression, anxiety, and alexithymia (5) abnormal food-related beliefs are associated with emotion regulation strategies and difficulties.

## Materials and methods

### Participants

The study involved 76 women aged 22 to 60 years. Thirty-five women were outpatients diagnosed with binge-eating disorder, assessed from January 2019 to April 2020. To be enrolled in the clinical group, the diagnosis had to be verified by the consultant psychiatrist (KK) based on a clinical interview and fulfilling DSM-5 criteria [1] for binge-eating disorder. To ensure a homogeneous sample, comorbid psychiatric diagnoses of personality disorders, bipolar affective disorder, psychoses, as well as habitual substance abuse were an exclusion factor as well as neurological disorders and pregnancy. The healthy control subjects consisted of 41 women recruited from a community setting, including university students and staff, properly matched to the age of the clinical group. They were recruited based upon clinical measures as well as clinical interviews according to DSM-5 to exclude the presence of any eating or other psychiatric disorders. Inclusion criteria for the healthy sample were: (1) normal body mass index (BMI) in the range from 18.5 to 24.9 kg/m^2^ and (2) no current diagnosis of any mental disorder according to DSM-5.

### Procedure

Recruitment took place in both outpatient obesity clinic and mental health outpatient settings in Warsaw, in local Anonymous Eaters Association, and via websites for people with binge-eating problems using a study project advert. Every participant signed the informed consent for participation in the study. There was no remuneration for participating in the study. The study was approved by the local ethics committee (Institutional Review Board; number 05/2019).

Recruited participants were asked to complete two questionnaires to verify the presence and severity of the eating disorders psychopathology, i.e. the Eating Attitudes Test (EAT-26 [34, 35];) and the Eating Beliefs Questionnaire (EBQ-18 [3];). The participants were then asked to provide their weight and height to calculate the BMI and to complete the Hospital Anxiety and Depression Scale (HADS [36, 37];), the Toronto Alexithymia Scale (TAS-20 [38, 39];), the Cognitive Emotion Regulation Questionnaire (CERQ [24, 40];), and the Difficulties in Emotion Regulation Scale (DERS [41];). The control group underwent the same test procedure.

#### Questionnaire measures

##### Eating Attitude Test (EAT-26)

Nutrition problems were first evaluated using the Eating Attitudes Test (EAT-26 [34]; Polish adaptation by Rogoza et al. [35]); scale reliability (α = 0.80). This is a widely used 26-item standardized self-report measurement, consisting of the following subscales: dieting, oral control, bulimia, and food preoccupation. Each item is rated on a 6-point scale ranging from 0 (never) to 6 (always). A score of above 20 suggests abnormal eating behaviors or a high level of concerns about weight and dieting.

##### Eating Beliefs Questionnaire (EBQ-18)

The Eating Beliefs Questionnaire (EBQ-18 [3]) (internal consistency α = 0.94), was used to measure negative, positive, and permissive beliefs about food, which, according to the authors of the tool [3] are the underlying factor of binge-eating. Participants provide a rating on a 5-point scale of how strongly they agree with the statement from 1 (strongly disagree) to 5 (strongly agree). Higher scores indicate increased severity in eating psychopathology. The tool in its original version has good test-retest reliability at an interval of 2 weeks (scale-dependent r ranging between 0.74 and 0.90).

##### Hospital Anxiety and Depression Scale (HADS)

The level of anxiety and depression symptoms was measured with the Hospital Anxiety and Depression Scale (HADS [36]; Polish validation by Nezlek [37]), which is a commonly used tool consisting of 14 items grouped into two subscales corresponding to the anxiety component (7 items) and the depression component (7 items). Each item is rated on a 4 point rating scale (0–3). The result can be calculated separately for each scale. The Polish version of the tool has very good internal consistency (α = 0.88) [42].

##### Toronto Alexithymia Scale (TAS-20)

The level of alexithymia was measured with the Toronto Alexithymia Scale (TAS-20 [38];; Polish adaptation by Ścigała et al. [39]) consisting of 20 items grouped into three subscales: Difficulty Identifying Feeling (DIF), Difficulty Describing Feeling (DDF) and Externally Oriented Thinking (EOT). Participants rate each item on a rating scale from 1 to 5, with a higher score indicating higher degrees of alexithymia. The Polish version of the tool has good psychometric properties (α = 0.82–0.86) [39].

##### Difficulties in Emotion Regulation Scale (DERS)

The evaluation of the regulation of emotions was performed with the Difficulties in Emotion Regulation Scale (DERS [41];; Polish translation by Czub [43]), which is a self-reporting tool consisting of 36 items that create the following dimensions: nonacceptance of emotional responses, difficulties engaging in goal-directed behavior, impulse control difficulties, limited access to emotion regulation strategies, lack of emotional awareness, and lack of emotional clarity. Questions are answered by specifying the frequency of the experience on a 5-point rating scale (0 never to 5 all the time). The English version of the tool has high internal consistency (α = 0.93) and good test-retest reliability over a period ranging from 4 to 8 weeks (ρI = .88, *p* < 0.01) [40].

##### Cognitive Emotion Regulation Questionnaire (CERQ)

The use of specific strategies, both adaptive and maladaptive, was studied using the Cognitive Emotion Regulation Questionnaire (CERQ [24];) in the Polish adaptation by Marszał-Wiśniewska and Fajkowska [40]. The adaptive strategies comprise acceptance, positive refocusing, refocusing on planning, positive reappraisal, and putting into perspective, while the maladaptive strategies include self-blame, other-blame, catastrophizing, and rumination. The complete tool consists of 36 items. Participants rate the frequency of the experience on 5-point rating scales (1 almost never to 5 almost always). The Polish version is characterized by good internal consistency (α for 8 out of 9 strategies is 0.70–0.87, and only for acceptance strategy it is 0.52) and satisfactory theoretical accuracy relating to the correlations between the strategy of emotional regulation and emotional, temperamental, and volitional properties [40].

### Statistical analyses

To test the hypotheses, statistical analyses were performed using IBM SPSS Statistics software version 25 (IBM, Armonk, NY). It was used to calculate basic descriptive statistics, normal distribution tests, and, depending on their results, t-tests or Mann–Whitney U tests for testing group differences. Correlation analyses both in the BED group and the healthy controls were performed using *Pearson’s r* coefficient or its non-parametric equivalent of *Spearman’s rho* in case of non-fullfilment of the requirements. To account for multiple comparisons with the problem of alpha-error inflation, we applied the Bonferroni correction.

## Results

### Sample characteristics

#### Sociodemographic variables

No statistically significant age difference was observed between the groups. There was a significant difference in BMI between subject groups indicating overweight on the verge of obesity in BED group and normal body weight in healthy control (Table 1).

**Table 1:** Sociodemographic data in both subject groups

	BED group *n* = 35		Healthy controls *n* = 41		t-test
	M	SD	M	SD	*p-value*
Age	33.60	9.71	37.51	13.85	.165
BMI	29.98	8.90	22.84	3.13	**< .001**

Notes: *BMI* Body Mass Index, *M* mean, *SD* standard deviation

#### Clinical variables

The student t-test showed that women in the clinical group were characterized by significantly higher levels of eating disorders symptoms (overall EAT-26 result and its subscales dieting and bulimia and food preoccupation), alexithymia (mean global score, difficulty in identifying feeling, difficulty in describing feeling), anxiety, and depressive symptoms than control group. Additionally, in the group with BED, a higher number of positive, negative and permissive food-related beliefs were observed. These differences were highly significant (*p* < 0.001), also in the case of the global score. The observed effect sizes for all variables were large (except the difficulty in describing feelings to others, with a medium effect size). Detailed values of the discussed analyses are presented in Table 2.

**Table 2:** The differences between the BED group and healthy controls in the EAT-26, EBQ-18, TAS-20, and HADS tool dimension levels

Variables	BED group		Healthy controls						
	*M*	*SD*	*M*	*SD*	*t*	*p*	*LL*	*UL*	*Cohen d*
EAT-26: Global score	25.75	12.34	5.82	5.39	8.50	**< 0.001**	15.19	24.67	2.09
Dieting	16.70	8.08	4.50	4.76	7.65	**< 0.001**	8.99	15.40	1.84
Bulimia and food preoccupation	7.09	3.24	0.38	1.10	11.37	**< 0.001**	5.52	7.90	2.77
Oral control	2.23	3.52	1.12	1.78	1.68	0.099	−0.21	2.43	0.40
EBQ-18: Global score	62.61	8.86	30.37	7.79	17.00	**< 0.001**	28.47	36.02	3.86
Positive beliefs	22.00	4.48	10.02	3.80	12.76	**< 0.001**	10.11	13.84	2.88
Negative beliefs	23.06	4.49	9.29	2.44	16.38	**< 0.001**	12.08	15.45	3.81
Permissive beliefs	17.56	4.21	11.48	4.03	6.50	**< 0.001**	4.22	7.94	1.48
Alexithymia	56.71	14.29	44.48	11.33	4.19	**< 0.001**	6.42	18.06	0.96
Difficulty identifying feeling	22.17	6.82	14.93	5.65	5.10	**< 0.001**	4.41	10.07	1.17
Difficulty describing feeling	15.14	5.44	11.86	4.08	3.04	**0.003**	1.13	5.43	0.69
Externally oriented thinking	19.39	4.96	17.69	4.09	1.66	0.101	−0.34	3.74	0.38
Anxiety	12.92	3.79	7.52	3.64	6.40	**< 0.001**	3.71	7.07	1.45
Depression	8.39	4.02	4.50	3.36	4.59	**< 0.001**	2.20	5.58	1.05

Notes: Cohen d – effect size measure; **bold**: statistically significant results after the Bonferroni correction (alpha level set to 0,05/14 ~ 0,00357)

*EAT-26* Eating Attitudes Test, *EBQ-18* Eating Beliefs Questionnaire-18, *TAS-20* Toronto Alexithymia Scale-20, *HADS* Hospital Anxiety and Depression Scale, *M* mean, *SD* standard deviation, *LL* lower limit of 95% confidence interval, *UL* upper limit of 95% confidence interval

### Emotion regulation

In the DERS, women in the clinical group were characterized by significantly higher levels of difficulties in emotion regulation, nonacceptance of emotional responses, difficulties engaging in goal-directed behavior, impulse control difficulties, limited access to emotion regulation strategies, and lack of emotional clarity than in the control group. Solely the subscale of lack of emotional awareness revealed non-significant differences. The magnitude of effects for all statistically significant differences were substantial. The exact values of the discussed analyses are presented in Table 3.

**Table 3:** The differences between the BED group and healthy controls in the DERS tool dimension levels

*Variables*	BED group		Healthy controls						
	*M*	*SD*	*M*	*SD*	*t*	*p*	*LL*	*UL*	Cohen *d*
DERS: Global score	110.68	25.33	82.43	18.71	5.38	**< 0.001**	17.74	38.76	1.28
Nonacceptance of emotional responses	18.08	7.03	12.79	4.53	3.88	**< 0.001**	2.57	8.03	0.91
Difficulties engaging in goal directed behavior	18.14	5.09	14.23	4.03	3.72	**< 0.001**	1.82	6.02	0.86
Impulse control difficulties	18.14	6.45	13.07	4.35	4.00	**< 0.001**	2.53	7.60	0.93
Lack of emotional awareness	16.83	5.05	16.38	4.42	0.42	0.674	−1.68	2.59	0.10
Limited access to emotion regulation strategies	25.14	7.78	17.05	5.43	5.19	**< 0.001**	4.98	11.21	1.23
Lack of emotional clarity	13.86	5.25	9.21	2.91	4.72	**< 0.001**	2.67	6.62	1.12

Notes: Cohen d – effect size measure; **bold**: statistically significant results after the Bonferroni correction (alpha level set to 0,05/7 ~ 0,0071)

*DERS* Difficulties in Emotion Regulation Scale, *M* mean, *SD* standard deviation, *LL* lower limit of 95% confidence interval, *UL* upper limit of 95% confidence interval

In the CERQ, women in the healthy control group had significantly higher levels of positive refocusing and putting into perspective than in the clinical group. On the other hand, women in the BED group were characterized by significantly higher levels of rumination and self-blame than those in the control group. The observed effect sizes measured by Cohen’s d coefficients ranged from 0.66 to 1.34, indicating moderate to strong effects. Detailed values of the discussed tests are presented in Table 4.

**Table 4:** The differences between the BED group and healthy controls in the CERQ tool dimension levels

*Variables*	BED group		Healthy controls						
	*M*	*SD*	*M*	*SD*	*t*	*p*	*LL*	*UL*	Cohen *d*
Acceptance	13.79	2.68	12.83	2.67	1.56	0.124	−0.27	2.19	0.36
Refocus on planning	13.17	3.64	14.95	2.65	−2.43	0.018	−3.25	−0.32	0.57
Positive refocusing	9.19	3.47	12.69	2.92	−4.83	**< 0.001**	− 4.94	− 2.06	1.10
Positive reappraisal	12.23	3.73	14.26	3.08	−2.62	0.011	−3.58	−0.49	0.60
Putting into perspective	11.91	3.48	14.02	2.98	−2.87	**0.005**	−3.58	−0.64	0.66
Self-blame	14.47	3.16	10.55	2.73	5.80	**< 0.001**	2.57	5.27	1.34
Other-blame	9.25	4.18	8.98	2.56	0.34	0.734	−1.34	1.88	0.08
Rumination	14.81	2.96	12.21	2.85	3.93	**< 0.001**	1.28	3.90	0.89
Catastrophizing	11.09	4.02	9.00	2.86	2.56	0.013	0.46	3.71	0.61

Notes: Cohen d – effect size measure; **bold**: statistically significant results after the Bonferroni correction (alpha level set to 0,05/9 ~ 0,0056)

*CERQ* Cognitive Emotion Regulation Questionnaire, *M* mean, *SD* standard deviation, *LL* lower limit of 95% confidence interval, *UL* upper limit of 95% confidence interval

### Correlation analyses

The analysis of the correlation in the clinical and control group between the DERS and CERQ dimensions and clinical variables using the Pearson’s r or non-parametric Spearman’s rho factor revealed the presence of moderately strong, strong, and very strong correlations. Due to the large size of the tables and the amount of statistically significant relations, all results can be found in the Appendix – Table A1, A2 (BED group), and Table A3, A4 (HC group).

#### Difficulties in emotion regulation scale

In the BED group, several statistically significant positive correlations between the subscales of DERS and the clinical variables were observed. Difficulties in emotion regulation were very strongly correlated with alexithymia (*p* < 0.001), and its subscale: difficulty in describing feeling (*p* < 0.001), and very strongly with difficulty in identifying feeling (*p* < 0.001). Also, strong relationships were detected between difficulties in emotion regulation and anxiety (*p* < 0.001), and depression (*p* < 0.001). Nonacceptance of emotional responses was found to be strongly correlated with alexithymia (*p* < 0.001) and its subscale: difficulty in describing feeling (*p* < 0.001), anxiety (*p* < 0.001), and depression (*p* < 0.001), as well as moderately with difficulty in identifying feeling (*p* = 0.004) (also an alexithymia subscale). There was a moderate correlation between difficulties engaging in goal directed behavior and intensity of depressive symptoms (*p* = 0.007). Impulse control difficulties were moderately correlated with alexithymia (*p* = 0.005) and its subscale: difficulty in identifying feeling (*p* = 0.007), and strongly with difficulty in describing feeling (*p* < 0.001). Lack of emotional awareness was found to be strongly correlated with alexithymia (*p* = 0.002). Strong relationships were also detected between limited access to emotion regulation strategies and alexithymia (*p* < 0.001) and its subscales: difficulty in identifying feeling (*p* = 0.001), and difficulty in describing feeling (*p* < 0.001), anxiety (*p* < 0.001), and depression (*p* < 0.001). Finally, lack of emotional clarity correlated very strongly with alexithymia (*p* < 0.001) and its subscales: difficulty in identifying feeling (*p* < 0.001) and difficulty in describing feeling (*p* < 0.001), and strongly with anxiety (*p* < 0.001).

In the healthy controls group, only three dimensions of the DERS were found to be significantly and positively correlated with clinical measures. Difficulties in emotion regulation were moderately correlated with difficulty in identifying feeling (*p* = 0.006), and moderately with intensity of depressive symptoms (*p* = 0.003). A moderate correlation was also found between nonacceptance of emotional responses and severity of depressive symptoms (*p* = 0.003). Finally, lack of emotional clarity was strongly correlated with alexithymia (*p* < 0.001) and its subscales: difficulty in identifying feeling (*p* < 0.001), and difficulty in describing feeling (*p* < 0.001), and with intensity of depressive symptoms (*p* < 0.001).

#### Cognitive emotion regulation questionnaire

In the BED group, a strong positive relationship was revealed between self-blame (CERQ subscale) and anxiety (*p* = 0.002). A strong positive correlation was also observed also between the catastrophizing subscale and intensity of eating disorders (i.e. the global EAT-26 score; *p* = 0.002, and its subscale dieting; *p* < 0.001). The correlation between catastrophizing and anxiety was moderate (*p* = 0.004).

In the healthy controls group, the only statistically significant correlation between the CERQ and the clinical variables was found between self-blame and externally oriented thinking (a moderate negative correlation, *p* = 0.005).

## Discussion

The main aim of this study was to evaluate strategies of emotional regulation in women struggling with binge-eating disorder. According to the main hypothesis raised, patients with BED were expected to experience more difficulties in this domain and to use more maladaptive emotion regulation strategies and fewer adaptive strategies than healthy women in the control group. Indeed, a higher nonacceptance of emotional responses, lack of emotional clarity, difficulties engaging in goal-directed behavior, impulse control difficulties, and limited access to emotion regulation strategies were observed in the BED group compared to healthy women. It should be noted that the effect was strong in each case. This is consistent with the literature reports highlighting deficits in these areas in patients with BED [7, 8, 17, 20, 32]. Interestingly, however, a difference was observed between the groups assessed in this study regarding the nonacceptance of emotional responses (DERS) subscale, whereas it was not observed in the acceptance subscale understood as acceptance of negative events (CERQ). This indicates the multifaceted construct which acceptance is. The lack of difficulties in agreeing to negative events may result from the presence of variables that were not considered in this study, such as internal attribution style or low self-assessment. They may influence the perception of negative events in life as completely natural, consistent with the negative way of thinking about oneself [44]. However, this observation would need to be verified in subsequent studies.

Greater level of impulse control difficulties seems to follow other studies [45, 46] reporting high *negative urgency* (the tendency to act rashly when distressed) in individuals with BED. These findings highlight the potential value of focusing on this facet of impulsivity, i.e. negative urgency in treatments for BED, as it may play a role between negative affect and binge-eating [32]. By assessing a wide range of emotion regulation strategies, this study showed that patients with BED use more maladaptive strategies to regulate emotions than healthy women, which is consistent with the current research (i.e. [29, 30]). Healthy controls applied positive refocusing and putting into perspective more often than patients with BED did. This is consistent with Kelly, Lydecker and Mazzeo’s [47] study results performed in a large group of individuals (*N* = 419). Above mentioned authors [47] demonstrated that the use of more positive strategies is associated with a lower frequency of binge-eating. When it comes to negative strategies, in the present study women from clinical group were more inclinated to use rumination and self-blame (strong effects) than healthy women. These two maladaptive strategies have been raised by other authors. For example, Svaldi et al. [8] noticed that in comparison to healthy women, BED patients’ self-blame more often. It might be related to the feeling of shame, which is characteristic for individuals with BED [1]. Patients with this disorder may be ashamed of e.g. their body weight or having no control over food intake, and perceive its as one’s own fault [1]. However, by far the strongest evidence in the research literature is provided for using rumination as a strategy by BED patients (i.e. [43, 22, 27, 28, 48]). In one longitudinal study conducted by Nolen-Hoeksem et al. [25], the use of this strategy by teenage girls even predicted the development of binge-eating in the future. Therefore, the search for further relationships between the applied strategies and clinical symptoms accompanying BED (i.e. severity of eating disorder, and symptoms of anxiety, depression, and alexithymia) seems justified.

In present study, many strong, statistically significant relationships were observed in the BED group between higher difficulties in emotion regulation (nonacceptance of emotional responses, impulse control difficulties, lack of emotional awareness, limited access to emotion regulation strategies, lack of emotional clarity), and higher level of alexithymia (and its subscales: difficulty in identifying and describing feelings). What is important, in the healthy controls, only one aspect (lack of emotional clarity) was significantly related to alexithymia. Therefore, one might hypothesize that problems in identifying and describing feelings may play an essential role in difficulties in emotion regulation observed in people with BED. Although this hypothesis needs to be verified in further research, some other studies appear supportive of it. For example, in a study of Brown et al. [49] conducted with patients with eating disorders (anorexia nervosa and bulimia nervosa), admission alexithymia was related to levels and change in global emotion dysregulation, access to emotion regulation strategies, and impulse control difficulties. Moreover, in the anorexia nervosa group, lower levels of alexithymia coexisted with lower levels of emotion dysregulation [49]. Also, the findings of Pandey et al. [50] showed that alexithymia was related to greater emotion regulation difficulties, which were additionally associated with severe health-related problems. There are no studies examining relationships between alexithymia and emotion regulation difficulties in BED individuals to the authors’ best knowledge.

The present study also revealed the associations between greater difficulties in emotion regulation in the BED group (nonacceptance of emotional responses, lack of emotional clarity, difficulties engaging in goal- directed behavior, limited access to emotion regulation strategies) and higher intensity of anxiety and depressive symptoms. Interestingly, in the healthy control group, this relationship was weaker and occurred only concerning the first two of the mentioned subscales. It might be hypothesized that anxiety and depression could be, at least, partially the consequence of difficulties in emotion regulation. This thesis could be supported by the results obtained by Gonçalves and colleagues [51]. In this cross-sectional and longitudinal study, the authors showed that overall difficulties in emotion regulation and limited access to emotion regulation strategies predict the intensity of depressive symptoms. Therefore, they recommend the inclusion of interventions aimed at improving emotional regulation in the prevention of depression. As far as anxiety is concerned, Menezes and colleagues [52] showed that improvement in emotion regulation processes in general emotion dysregulation and difficulties in regulatory strategies after 6 weeks of training was related to reduction of trait anxiety. In other studies, associations between lower difficulties in emotion regulation and lower anxiety in patients with BED were also confirmed [53, 54]. However, after Bonferroni correction, no statistically significant correlations remained in the clinical group between eating disorders psychopathology and difficulties in emotion regulation. Considering that before the application of the Bonferroni correction, the vast majority of the mentioned correlations exceed the threshold of statistical significance (*p* < 0.05), one might hypothesize that in the case of a larger number of participants, these relationships would remain significant also after Bonferroni correction. Additionally, both depressive and anxiety symptoms are constructs that focus to a greater extent on emotional aspects of functioning than in the case of eating disorders psychopathology which is more multidimensional (very important role of behavioral components). That could make it understandable that the correlations between anxiety and depressive symptoms and emotion regulation become more pronounced. It also seems possible that anxiety and/or depressive symptoms could potentially be mediators / moderators of the relationship between emotion regulation and eating disorders psychopathology. However, further research with a greater number of participants is necessary to verify those hypotheses and deepen the knowledge about above mentioned relationships.

When it comes to using strategies of emotion regulation by individuals with BED, the present study revealed that the higher level of anxiety was related to higher self-blame and catastrophizing. The latter was also associated with a higher level of eating disorders pathology. Interestingly, those dependencies were not observed in healthy controls, suggesting that they are specific for psychopathology accompanying BED. It is possible that - as it was obtained in a study by Martin and Dahlen [55] - catastrophizing and self-blame would be factors that enable the prediction of anxiety symptoms. If so, decreasing maladaptive and developing new, adaptive strategies of emotion regulation would be an important factor in dealing with extended anxiety in people with BED. However, further research is necessary to explore the direction of these relationships. It is especially important in relation to catastrophizing as it was the only emotion regulation strategy strongly related to the severity of eating disorder symptoms.

### Limitations and future directions

When considering the study results, some caution should be taken about the limitations which, despite efforts, could not be avoided. The final size of the clinical group due to the desire to gather a homogeneous group of patients with BED is relatively small. However, the effect sizes were mostly substantial; thus, our study seems sufficiently powered. Additionally, the correlational nature of the study does not allow for drawing conclusions about causality, but only for creating hypotheses. Further research using a larger sample is necessary to enable more advanced statistical analyses, which could be very interesting especially in reference to the alexithymia construct. Inclusion of men in the study would also allow conclusions to be drawn about the differences between the sexes in the clinical picture of BED. As this study is mainly based on retrospective self-report measures with the possibility of recall biases, it also seems appropriate to design studies using methods that eliminate those, e.g. e-diary studies, and studies conducted in the natural environment of BED individuals (i.e. by Ecological Sampling Method). Finally, since the changes in emotions are of a dynamic nature, it would be important, apart from cross-sectional studies, to conduct longitudinal and prospective studies that would enable the observation of this phenomenon.

## Conclusions

Overall, the results obtained in this study are consistent with previous findings on the complex BED psychopathology, indicating an important and still insufficiently understood role of emotional dysregulation in the clinical picture of this disorder. Several significant relationships between various difficulties in emotion regulation and alexithymia suggests the importance of this construct and support the increasingly popular position that therapeutic work with people struggling with BED should be aimed not only at difficulties in emotion regulation themselves, but first of all at more basic abilities – i.e. recognizing and naming emotions, increasing their clarity, and learning to accept them [56]. Among the analyzed strategies, only catastrophizing and self-blame were related to the psychopathology observed in the BED group while solely catastrophizing turned out to be related to symptoms of eating disorders. Therefore, it might be beneficial to pay special attention to them while planning therapeutic interventions in BED.

## Supplementary Information

**Additional file 1: Table A1.** Correlation between the Difficulties in Emotion Regulation Scale (DERS) dimensions and clinical variables in the BED group. **Table A2.** Correlation between the Cognitive Emotion Regulation Questionnaire (CERQ) dimensions and clinical variables in the BED group. **Table A3.** Correlation between the Difficulties in Emotion Regulation Scale (DERS) dimensions and clinical variables in the control group. **Table A4.** Correlation between the Cognitive Emotion Regulation Questionnaire (CERQ) dimensions and clinical variables in the control group.

## Data Availability

Data access can be requested from the corresponding author on reasonable demand.
