# Supplementary material for: Examining emotion regulation in binge-eating disorder
Source: Borderline Personal Disord Emot Dysregul. 2021 Oct 8;8:25. doi: 10.1186/s40479-021-00166-6 (PMC8504023; doi:10.1186/s40479-021-00166-6)
Supplement: Supplementary file 1 — Additional file 1: Table A1. Correlation between the Difficulties in Emotion Regulation Scale (DERS) dimensions and clinical variables in the BED group. Table A2. Correlation between the Cognitive Emotion Regulation Questionnaire (CERQ) dimensions and clinical variables in the BED group. Table A3. Correlation between the Difficulties in Emotion Regulation Scale (DERS) dimensions and clinical variables in the control group. Table A4. Correlation between the Cognitive Emotion Regulation Questionnaire (CERQ) dimensions and clinical variables in the control group. [file 40479_2021_166_MOESM1_ESM.docx]

| **Table A1. Correlation between the Difficulties in Emotion Regulation Scale (DERS) dimensions and clinical variables in the BED group** | | | | | | | | |
| --- | --- | --- | --- | --- | --- | --- | --- | --- |
| Variables | Indicators | **Difficulties in Emotion Regulation Scale (DERS)** | | | | | | |
|  |  | Difficulties  in Emotion Regulation Scale | Nonacceptance of emotional responses | Difficulties engaging in goal directed behaviour | Impulse control difficulties | Lack of emotional awareness | Limited access to emotion regulation strategies | Lack of emotional clarity |
| Alexithymia | *r* Pearson | **0,71*** | **0,53*** | 0,27 | **0,46*** | **0,50*** | **0,55*** | **0,79*** |
|  | *p* | **<0,001** | **0,001** | 0,116 | **0,005** | **0,002** | **<0,001** | **<0,001** |
| Difficulty in identifying feeling | *r* Pearson | **0,64*** | **0,48*** | 0,25 | **0,44*** | 0,40 | **0,54*** | **0,70*** |
|  | *p* | **<0,001** | **0,004** | 0,153 | **0,007** | 0,018 | **0,001** | **<0,001** |
| Difficulty in describing feeling | *r* Pearson | **0,73*** | **0,58*** | 0,30 | **0,51*** | 0,39 | **0,59*** | **0,79*** |
|  | *p* | **<0,001** | **<0,001** | 0,083 | **0,001** | 0,017 | **<0,001** | **<0,001** |
| Externally oriented thinking | *r* Pearson | 0,33 | 0,21 | 0,12 | 0,13 | 0,45 | 0,18 | 0,40 |
|  | *p* | 0,060 | 0,221 | 0,507 | 0,452 | 0,005 | 0,304 | 0,016 |
| Anxiety | *r* Pearson | **0,59*** | **0,52*** | 0,22 | 0,41 | 0,08 | **0,62*** | **0,57*** |
|  | *p* | **<0,001** | **0,001** | 0,202 | 0,013 | 0,625 | **<0,001** | **<0,001** |
| Depression | *r* Pearson | **0,56*** | **0,52*** | **0,45*** | 0,24 | 0,10 | **0,55*** | 0,32 |
|  | *p* | **<0,001** | **0,001** | **0,007** | 0,159 | 0,572 | **<0,001** | 0,054 |
| Global score EAT-26 | *r* Pearson | 0,38 | 0,37 | 0,29 | 0,36 | -0,16 | 0,45 | 0,40 |
|  | *p* | 0,037 | 0,037 | 0,120 | 0,041 | 0,392 | 0,010 | 0,025 |
| Dieting | *r* Pearson | 0,34 | 0,37 | 0,35 | 0,20 | -0,10 | 0,38 | 0,35 |
|  | *p* | 0,058 | 0,033 | 0,051 | 0,258 | 0,580 | 0,030 | 0,049 |
| Bulimia and food preoccupation | *r* Pearson | 0,28 | 0,23 | 0,14 | 0,35 | -0,15 | 0,41 | 0,33 |
|  | *p* | 0,123 | 0,191 | 0,449 | 0,044 | 0,402 | 0,020 | 0,064 |
| Oral control | *rho Spearman* | 0,32 | 0,25 | 0,15 | 0,36 | -0,12 | 0,37 | 0,33 |
|  | *p* | 0,070 | 0,149 | 0,396 | 0,034 | 0,494 | 0,034 | 0,055 |
| Global score EBQ-18 | *r* Pearson | 0,26 | 0,37 | 0,08 | 0,23 | -0,09 | 0,27 | 0,04 |
|  | *p* | 0,132 | 0,026 | 0,644 | 0,170 | 0,614 | 0,116 | 0,798 |
| Negative beliefs | *r* Pearson | 0,31 | 0,32 | -0,06 | 0,35 | 0,11 | 0,30 | 0,31 |
|  | *p* | 0,074 | 0,057 | 0,730 | 0,039 | 0,538 | 0,079 | 0,064 |
| Positive beliefs | *r* Pearson | 0,07 | 0,26 | 0,12 | 0,07 | -0,14 | 0,07 | -0,21 |
|  | *p* | 0,685 | 0,127 | 0,483 | 0,693 | 0,407 | 0,699 | 0,209 |
| Permissive beliefs | *r* Pearson | 0,16 | 0,16 | 0,10 | 0,05 | -0,14 | 0,19 | -0,01 |
|  | *p* | 0,374 | 0,350 | 0,550 | 0,769 | 0,400 | 0,285 | 0,951 |

**Notes:** EAT-26 – Eating Attitudes Test; EBQ-18 – Eating Beliefs Questionnaire-18; ***bold:** statistically significant results after the Bonferroni correction (alpha level

set to 0,05/7 ~ 0,0071 for DERS)

| **Table A2. Correlation between the Cognitive Emotion Regulation Questionnaire (CERQ) dimensions and clinical variables in the BED group** | | | | | | | | | | |
| --- | --- | --- | --- | --- | --- | --- | --- | --- | --- | --- |
| Variables | Indicators | **Cognitive Emotion Regulation Questionnaire (CERQ)** | | | | | | | | |
|  |  | Acceptance | Refocus on planning | Positive refocusing*.* | Positive reappraisal | Putting into perspective | Self-blame | Other- blame | Rumination | Catastrophizing |
| Alexithymia | *r* Pearson | -0,12 | -0,31 | -0,35 | -0,14 | 0,06 | 0,09 | 0,39 | -0,01 | 0,39 |
|  | *p* | 0,494 | 0,068 | 0,040 | 0,446 | 0,720 | 0,610 | 0,021 | 0,955 | 0,024 |
| Difficulty in identifying feeling | *r* Pearson | 0,00 | -0,27 | -0,37 | -0,25 | -0,01 | 0,11 | 0,31 | 0,05 | 0,35 |
|  | *p* | 0,994 | 0,120 | 0,029 | 0,154 | 0,933 | 0,558 | 0,070 | 0,793 | 0,041 |
| Difficulty in describing feeling | *r* Pearson | -0,13 | -0,29 | -0,34 | -0,06 | 0,09 | 0,09 | 0,39 | 0,05 | 0,41 |
|  | *p* | 0,481 | 0,085 | 0,040 | 0,729 | 0,623 | 0,614 | 0,018 | 0,751 | 0,015 |
| Externally oriented thinking | *r* Pearson | -0,21 | -0,20 | -0,10 | 0,03 | 0,10 | 0,02 | 0,25 | -0,15 | 0,17 |
|  | *p* | 0,239 | 0,243 | 0,559 | 0,873 | 0,570 | 0,894 | 0,135 | 0,395 | 0,340 |
| Anxiety | *r* Pearson | 0,03 | -0,23 | -0,26 | -0,28 | -0,05 | **0,51*** | 0,16 | 0,27 | **0,47*** |
|  | *p* | 0,852 | 0,172 | 0,130 | 0,099 | 0,760 | **0,002** | 0,352 | 0,107 | **0,004** |
| Depression | *r* Pearson | 0,03 | -0,35 | -0,28 | -0,39 | -0,17 | 0,36 | 0,20 | 0,17 | 0,34 |
|  | *p* | 0,865 | 0,038 | 0,093 | 0,020 | 0,316 | 0,035 | 0,244 | 0,330 | 0,044 |
| Global score EAT-26 | *r* Pearson | 0,18 | 0,09 | -0,04 | -0,07 | 0,16 | 0,26 | 0,27 | 0,42 | **0,52*** |
|  | *p* | 0,330 | 0,619 | 0,840 | 0,717 | 0,405 | 0,165 | 0,132 | 0,017 | **0,002** |
| Dieting | *r* Pearson | 0,03 | -0,01 | -0,07 | -0,17 | 0,01 | 0,14 | 0,30 | 0,43 | **0,61*** |
|  | *p* | 0,874 | 0,954 | 0,704 | 0,363 | 0,956 | 0,457 | 0,094 | 0,014 | **<0,001** |
| Bulimia and food preoccupation | *r* Pearson | 0,02 | 0,02 | -0,08 | 0,01 | 0,08 | 0,35 | -0,23 | 0,10 | 0,12 |
|  | *p* | 0,897 | 0,900 | 0,678 | 0,966 | 0,663 | 0,049 | 0,202 | 0,598 | 0,502 |
| Oral control | *rho Spearman* | 0,24 | 0,22 | -0,06 | -0,04 | 0,13 | 0,18 | **0,53*** | 0,41 | 0,42 |
|  | *p* | 0,173 | 0,201 | 0,713 | 0,811 | 0,478 | 0,313 | **0,001** | 0,014 | 0,013 |
| Global score EBQ-18 | *r* Pearson | 0,12 | -0,25 | -0,01 | -0,15 | -0,05 | 0,38 | -0,29 | 0,26 | 0,10 |
|  | *p* | 0,484 | 0,141 | 0,973 | 0,402 | 0,793 | 0,028 | 0,090 | 0,122 | 0,578 |
| Negative beliefs | *r* Pearson | 0,22 | -0,22 | -0,12 | -0,02 | 0,22 | 0,44 | -0,45 | -0,08 | -0,05 |
|  | *p* | 0,206 | 0,204 | 0,473 | 0,902 | 0,204 | 0,009 | 0,006 | 0,640 | 0,767 |
| Positive beliefs | *r* Pearson | -0,06 | -0,20 | 0,03 | -0,25 | -0,21 | 0,21 | -0,26 | 0,33 | 0,09 |
|  | *p* | 0,728 | 0,236 | 0,865 | 0,147 | 0,216 | 0,227 | 0,132 | 0,049 | 0,607 |
| Permissive beliefs | *r* Pearson | 0,09 | -0,08 | 0,09 | -0,02 | -0,11 | 0,11 | 0,15 | 0,29 | 0,17 |
|  | *p* | 0,606 | 0,648 | 0,609 | 0,922 | 0,531 | 0,529 | 0,396 | 0,607 | 0,343 |

**Notes:** EAT-26 – Eating Attitudes Test; EBQ-18 – Eating Beliefs Questionnaire-18; ***bold:** statistically significant results after the Bonferroni correction (alpha level set to 0,05/9 ~

0,0056 for CERQ)

| **Table A3. Correlation between the Difficulties in Emotion Regulation Scale (DERS) dimensions and clinical variables in the control group** | | | | | | | | |
| --- | --- | --- | --- | --- | --- | --- | --- | --- |
| Variables | Indicators | **Difficulties in Emotion Regulation Scale (DERS)** | | | | | | |
|  |  | Difficulties  in Emotion Regulation Scale | Nonacceptance of emotional responses | Difficulties engaging in goal directed behaviour | Impulse control difficulties | Lack of emotional awareness | Limited access to emotion regulation strategies | Lack of emotional clarity |
| Alexithymia | *r* Pearson | 0,36 | 0,27 | 0,19 | 0,21 | 0,20 | 0,20 | **0,61*** |
|  | *p* | 0,021 | 0,083 | 0,240 | 0,186 | 0,201 | 0,197 | **<0,001** |
| Difficulty in identifying feeling | *r* Pearson | **0,43*** | 0,39 | 0,25 | 0,19 | 0,11 | 0,35 | **0,62*** |
|  | *p* | **0,006** | 0,010 | 0,113 | 0,223 | 0,489 | 0,023 | **<0,001** |
| Difficulty in describing feeling | *r* Pearson | 0,28 | 0,23 | 0,16 | 0,18 | 0,10 | 0,14 | **0,56*** |
|  | *p* | 0,077 | 0,142 | 0,313 | 0,267 | 0,549 | 0,361 | **<0,001** |
| Externally oriented thinking | *r* Pearson | 0,13 | -0,02 | 0,01 | 0,14 | 0,31 | -0,07 | 0,28 |
|  | *p* | 0,424 | 0,890 | 0,955 | 0,386 | 0,044 | 0,682 | 0,074 |
| Anxiety | *r* Pearson | 0,28 | 0,34 | 0,17 | 0,11 | -0,03 | 0,29 | 0,38 |
|  | *p* | 0,085 | 0,030 | 0,302 | 0,482 | 0,869 | 0,063 | 0,014 |
| Depression | *r* Pearson | **0,47*** | **0,46*** | 0,20 | 0,28 | 0,16 | 0,38 | **0,62*** |
|  | *p* | **0,003** | **0,003** | 0,223 | 0,078 | 0,327 | 0,014 | **<0,001** |
| Global score EAT-26 | *r* Pearson | -0,11 | -0,11 | -0,11 | -0,02 | -0,17 | -0,05 | -0,02 |
|  | *p* | 0,510 | 0,494 | 0,526 | 0,915 | 0,288 | 0,765 | 0,914 |
| Dieting | *r* Pearson | -0,15 | -0,18 | -0,20 | -0,04 | -0,12 | -0,12 | 0,00 |
|  | *p* | 0,361 | 0,259 | 0,226 | 0,784 | 0,472 | 0,453 | 0,977 |
| Bulimia and food preoccupation | *r* Pearson | -0,06 | -0,11 | 0,01 | -0,19 | 0,08 | -0,12 | 0,11 |
|  | *p* | 0,700 | 0,507 | 0,961 | 0,218 | 0,594 | 0,445 | 0,485 |
| Oral control | *rho Spearman* | 0,15 | 0,22 | 0,31 | 0,10 | -0,24 | 0,19 | -0,07 |
|  | *p* | 0,360 | 0,172 | 0,055 | 0,515 | 0,131 | 0,222 | 0,657 |
| Global score EBQ-18 | *r* Pearson | -0,07 | -0,10 | 0,17 | -0,10 | -0,04 | -0,11 | -0,04 |
|  | *p* | 0,671 | 0,527 | 0,312 | 0,528 | 0,788 | 0,505 | 0,810 |
| Negative beliefs | *r* Pearson | -0,13 | -0,18 | -0,11 | -0,04 | 0,11 | -0,08 | -0,24 |
|  | *p* | 0,424 | 0,270 | 0,498 | 0,819 | 0,503 | 0,617 | 0,126 |
| Positive beliefs | *r* Pearson | -0,05 | -0,09 | 0,19 | -0,11 | -0,16 | -0,04 | 0,01 |
|  | *p* | 0,749 | 0,576 | 0,232 | 0,503 | 0,306 | 0,824 | 0,936 |
| Permissive beliefs | *r* Pearson | 0,03 | 0,05 | 0,22 | -0,07 | 0,05 | -0,16 | 0,20 |
|  | *p* | 0,845 | 0,761 | 0,169 | 0,646 | 0,774 | 0,300 | 0,206 |

**Notes:** EAT-26 – Eating Attitudes Test; EBQ-18 – Eating Beliefs Questionnaire-18; ***bold:** statistically significant results after the Bonferroni correction (alpha level

set to 0,05/7 ~ 0,0071 for DERS)

| **Table A4. Correlation between the Cognitive Emotion Regulation Questionnaire (CERQ) dimensions and clinical variables in the control group** | | | | | | | | | | |
| --- | --- | --- | --- | --- | --- | --- | --- | --- | --- | --- |
| Variables | Indicators | **Cognitive Emotion Regulation Questionnaire (CERQ)** | | | | | | | | |
|  |  | Acceptance | Refocus on planning | Positive refocusing*.* | Positive reappraisal | Putting into perspective | Self-blame | Other- blame | Rumination | Catastrophizing |
| Alexithymia | *r* Pearson | -0,14 | -0,33 | 0,02 | -0,37 | -0,22 | -0,10 | -0,07 | -0,07 | 0,01 |
|  | *p* | 0,362 | 0,033 | 0,896 | 0,016 | 0,168 | 0,509 | 0,666 | 0,647 | 0,970 |
| Difficulty in identifying feeling | *r* Pearson | 0,10 | -0,17 | 0,01 | -0,22 | -0,14 | 0,15 | 0,09 | 0,18 | 0,07 |
|  | *p* | 0,510 | 0,296 | 0,925 | 0,155 | 0,369 | 0,344 | 0,585 | 0,263 | 0,662 |
| Difficulty in describing feeling | *r* Pearson | -0,20 | -0,31 | 0,02 | -0,31 | -0,14 | -0,08 | -0,16 | -0,03 | -0,10 |
|  | *p* | 0,205 | 0,048 | 0,916 | 0,047 | 0,361 | 0,632 | 0,322 | 0,856 | 0,543 |
| Externally oriented thinking | *r* Pearson | -0,34 | -0,39 | 0,02 | -0,41 | -0,26 | **-0,42*** | -0,15 | -0,42 | 0,01 |
|  | *p* | 0,025 | 0,012 | 0,898 | 0,007 | 0,096 | **0,005** | 0,337 | 0,006 | 0,927 |
| Anxiety | *r* Pearson | 0,33 | 0,02 | -0,14 | -0,33 | -0,26 | 0,16 | 0,29 | 0,24 | 0,34 |
|  | *p* | 0,034 | 0,909 | 0,375 | 0,032 | 0,097 | 0,300 | 0,061 | 0,118 | 0,028 |
| Depression | *r* Pearson | 0,14 | -0,04 | -0,02 | -0,24 | 0,00 | 0,22 | 0,14 | 0,20 | 0,20 |
|  | *p* | 0,399 | 0,806 | 0,887 | 0,131 | 0,994 | 0,168 | 0,394 | 0,209 | 0,220 |
| Global score EAT-26 | *r* Pearson | 0,09 | 0,14 | -0,32 | 0,09 | 0,05 | 0,08 | 0,15 | 0,09 | -0,10 |
|  | *p* | 0,565 | 0,386 | 0,045 | 0,577 | 0,780 | 0,636 | 0,382 | 0,588 | 0,560 |
| Dieting | *r* Pearson | 0,04 | 0,12 | -0,22 | 0,15 | 0,16 | 0,08 | 0,07 | 0,02 | -0,05 |
|  | *p* | 0,819 | 0,462 | 0,172 | 0,342 | 0,339 | 0,620 | 0,690 | 0,920 | 0,767 |
| Bulimia and food preoccupation | *r* Pearson | 0,19 | 0,03 | 0,20 | 0,00 | -0,11 | -0,28 | -0,06 | -0,23 | 0,20 |
|  | *p* | 0,234 | 0,842 | 0,195 | 0,993 | 0,501 | 0,071 | 0,720 | 0,146 | 0,220 |
| Oral control | *rho Spearman* | 0,11 | 0,14 | -0,39 | -0,06 | -0,20 | 0,13 | 0,31 | 0,37 | 0,01 |
|  | *p* | 0,511 | 0,378 | 0,011 | 0,691 | 0,207 | 0,406 | 0,053 | 0,019 | 0,962 |
| Global score EBQ-18 | *r* Pearson | -0,05 | -0,07 | -0,09 | 0,15 | 0,22 | -0,08 | 0,10 | 0,10 | 0,05 |
|  | *p* | 0,779 | 0,686 | 0,593 | 0,355 | 0,165 | 0,640 | 0,548 | 0,533 | 0,771 |
| Negative beliefs | *r* Pearson | -0,16 | -0,12 | -0,16 | 0,12 | 0,27 | -0,21 | 0,02 | -0,08 | -0,01 |
|  | *p* | 0,312 | 0,451 | 0,319 | 0,442 | 0,085 | 0,183 | 0,883 | 0,622 | 0,930 |
| Positive beliefs | *r* Pearson | 0,09 | -0,07 | -0,04 | 0,07 | 0,19 | 0,03 | -0,05 | 0,18 | 0,05 |
|  | *p* | 0,563 | 0,645 | 0,796 | 0,639 | 0,229 | 0,854 | 0,777 | 0,268 | 0,753 |
| Permissive beliefs | *r* Pearson | 0,01 | 0,02 | 0,02 | 0,09 | 0,11 | 0,01 | 0,17 | 0,10 | 0,05 |
|  | *p* | 0,939 | 0,905 | 0,905 | 0,554 | 0,469 | 0,933 | 0,278 | 0,515 | 0,746 |

**Notes:** EAT-26 – Eating Attitudes Test; EBQ-18 – Eating Beliefs Questionnaire-18; ***bold:** statistically significant results after the Bonferroni correction (alpha level set to 0,05/9 ~

0,0056 for CERQ)
